# Can Museums Help Visitors Thrive? Review of Studies on Psychological Wellbeing in Museums

**DOI:** 10.3390/bs12110458

**Published:** 2022-11-17

**Authors:** Marta Šveb Dragija, Daniela Angelina Jelinčić

**Affiliations:** Institute for Development and International Relations, 10000 Zagreb, Croatia

**Keywords:** psychological wellbeing, wellbeing, museum, experience design

## Abstract

Museums are expected to prove their social value and ability to have a long-term social impact. Hence, in order to do so, museums, as experience hubs and the most-visited cultural attraction, may use their potential to offer experiences that could help visitors thrive by increasing their psychological wellbeing. Although psychological wellbeing has been a hot topic, the synthesized and holistic review of the literature on this theme has been lacking in regard to museums. Hence, we conducted an analysis using the PRISMA protocol to answer two research questions: (1) Can museums increase the visitor’s psychological wellbeing? (2) How can the museum experience be designed to enhance the psychological wellbeing of the visitors and how can that potentially be measured? The results showed that museums can enhance visitors’ and other stakeholders’ psychological wellbeing. This can be achieved by designing museum experiences that are attractive, comfortable (restorative), comprehensible, participative, innovative, and sustainable, relying on specific detailed guidelines provided in the article. The Museum Wellbeing Toolkit serves to measure the efficiency of the proposed guidelines in stimulating the psychological wellbeing of museum visitors. If backed by wellbeing policy frameworks, museums may increase their role in fostering psychological wellbeing. As wellbeing public policies have been rather rare to date, future research may explore the effects of the existing ones to provide recommendations for new developments on the topic.

## 1. Introduction

Museums’ role in society is changing and museums are expected to prove that they make a difference in terms of long-term social impact. Hence, museums across the world are shifting their attitudes from being centres for learning to also being centres for experiencing, which is opening the doors for the design of experiences that may enhance visitors’ lives by helping them thrive. The concept of experiences, as an economic offering, refers to memorable, out of the ordinary events that are customized for each specific consumer [1]. In museums, which are the most-visited cultural attraction across the world [2], such experiences can be visual, sensory, aesthetic, recreational, sociable, educational, celebrating and enchanting [3]. As the most-visited cultural attraction in the world, museums are a community resource, with an important role in providing educational leisure activities for the society, as well as providing opportunities for social interaction and participation. Furthermore, museums are highly valued institutions, and enjoy public trust (as well as subsidies in many cases), which puts them in a strong position to tackle challenging issues as well as offering them a platform to change people’s lives in a positive way. Subsequently, “Understanding these perceived benefits is therefore important both in meeting visitors’ needs, and in demonstrating the contribution that museums and other cultural attractions can make in adding perspective and meaning to people’s lives” [4] (p. 36). Thus, wellbeing has been recently increasingly recognized as an important museum experience outcome that also proves museums’ social value [5].

We may distinguish between subjective (or hedonic) and psychological (or eudemonic) wellbeing. The first type was proposed by Diener in 1984 and entails life satisfaction and positive emotions. According to this perspective, a person is well when he/she experiences more positive emotions and thoughts than negative ones [6]. On the other hand, psychological wellbeing, proposed by Ryff in 1989, is a more complex construct that involves the development and self-realization of the individual [7]. In other words, psychological wellbeing goes beyond a momentary experience of happiness and/or pleasure, and is “…the combination of feeling good and functioning well; the experience of positive emotions such as happiness and contentment as well as the development of one’s potential, having some control over one’s life, having a sense of purpose, and experiencing positive relationships” [8] (p. 1). This type of wellbeing has the capacity to change individual’s life because it entails personal growth, self-fulfilment and self-development, full engagement, and the optimal performance of meaningful behaviour [9]. Hence, in contrast to subjective wellbeing experiences, which are focused on positive emotions, museums that stimulate psychological wellbeing may use negative emotions (e.g., the Museum of Holocaust) that may challenge visitors to think about themselves and the world, hence propelling their personal growth [7]. Therefore, when museums offer experiences that can stimulate such changes, they move from “…collecting, preserving, and exhibiting objects, and educating the public, to understanding and meeting visitor’s multiple needs” [4] (p. 1). Moreover, by doing so, museums engage in the economy of wellbeing, which puts the wellbeing of people and planet at the centre of policy and decision-making [10]. 

To date, museums have mostly been studied regarding their educational benefits or as a hedonic, pleasure-driven activity that offers an immediate experience of positive emotions and has short-term effects that occur after involvement in the activity (such as happiness, pleasure, and satisfaction [11]. However, Pine and Gilmore suggested that experiences, and, thus, also experiences in museums, should yield transformations because “…the individual partaking in the experience often wants something more lasting than a memory, something beyond what any good, service or experience alone can offer” [12] (p. 39). According to them, these transformations should propel the visitor’s personal growth and psychological wellbeing. Furthermore, back in 2016, The European Travel Commission posited that travelling would soon be defined by people’s need to improve themselves and their lives; hence, the social value of museums, as providers of experiences (for locals and tourists) capable of enhancing visitor’s psychological wellbeing, became even more important [13].

The idea that museum’s social value lies in enhancing health and wellbeing is not new. Researchers have long been aware that museums may offer a restorative environment capable of reducing the mental exhaustion of visitors [4,14,15], and may lower blood pressure and stress [16], and increase visitors’ subjective wellbeing [17]. They offer an abundance of positive benefits for visitors’ personal growth, such as an increase in self-esteem, sense of identity, reduced social isolation and decreased anxiety [18,19]. However, to the best of our knowledge, although there is a summary of studies describing the benefits of museum activities in healthcare contexts [19], there is no systematic review of studies covering psychological wellbeing as a benefit of museum visitation for the general public.

Therefore, this study systematically explores and synthesizes the extant literature on psychological wellbeing as afforded by museum visits, with the goal of obtaining a holistic understanding of the field. By looking at the literature on psychological wellbeing in museums, this study places an emphasis on identifying, characterising, and summarising the current understanding of museums’ benefits for the psychological wellbeing of visitors. It also tries to identify the major gaps in the extant literature to shed light on potential directions for future studies. 

Understanding whether psychological wellbeing can be enhanced through museum visitation and how will enable us to offer practical guidelines for museum experience designers to create transformational and restorative experiences that can ensure the museums’ social value and their contribution to the community, which is important for the visitors and the museums’ sustainability. 

## 2. Aim of Study

Due to the lack of a systematic review of studies covering psychological wellbeing as a benefit of museum visitation for the general public of visitors, the aim of this study was to explore the literature on psychological wellbeing as afforded by museum visitation in order to synthesize the knowledge, identify gaps, and offer practical recommendations for the museum experience designers. To do so, the following research questions were posed:

1. Can museums increase the visitor’s psychological wellbeing?
2. How can the museum experience be designed to enhance the psychological wellbeing of the visitors and how can that potentially be measured?

The structure of the paper is as follows: first, the materials and methods used for the research review are explained. Then, the results, divided into several themes, are presented. Further, the results are discussed in relation to the research questions. Finally, directions for future studies are given, as well as guidelines for the museum experience designers. 

## 3. Materials and Methods

A systematic review of the selected academic and professional resources was carried out with the aim of presenting the literature on psychological wellbeing in museums. The literature search was conducted in September 2022, and all the articles published until September 2022 were included in the research to present an up-to-date review. Our search included original research papers, published in journals and book chapters written in English, obtained from a search of libraries worldwide using SmartCat. SmartCat is a research engine that searches libraries worldwide and includes databases such as Scopus, Web of Science, ProQuest, PsycINFO, and Science Direct. Articles and book chapters containing the following keywords were used in the search: (‘psychological wellbeing *’ OR ‘eudemonia *’ OR ‘wellbeing *’ OR ‘mental health *’ OR ‘restorative *’) AND (‘museum *’). We opted for including all these terms instead of only “psychological wellbeing” because the Boolean search which included (‘psychological wellbeing *’) AND (‘museum *’) yielded only four results. Since the mentioned terms are often used interchangeably, we included these in the analysis, expecting to find more results that, although not explicitly stated, still cover psychological wellbeing. The collected papers were then assessed based on their abstracts, and full texts when applicable. 

Our search found 78 results.

### Study Selection

**Figure 1:**
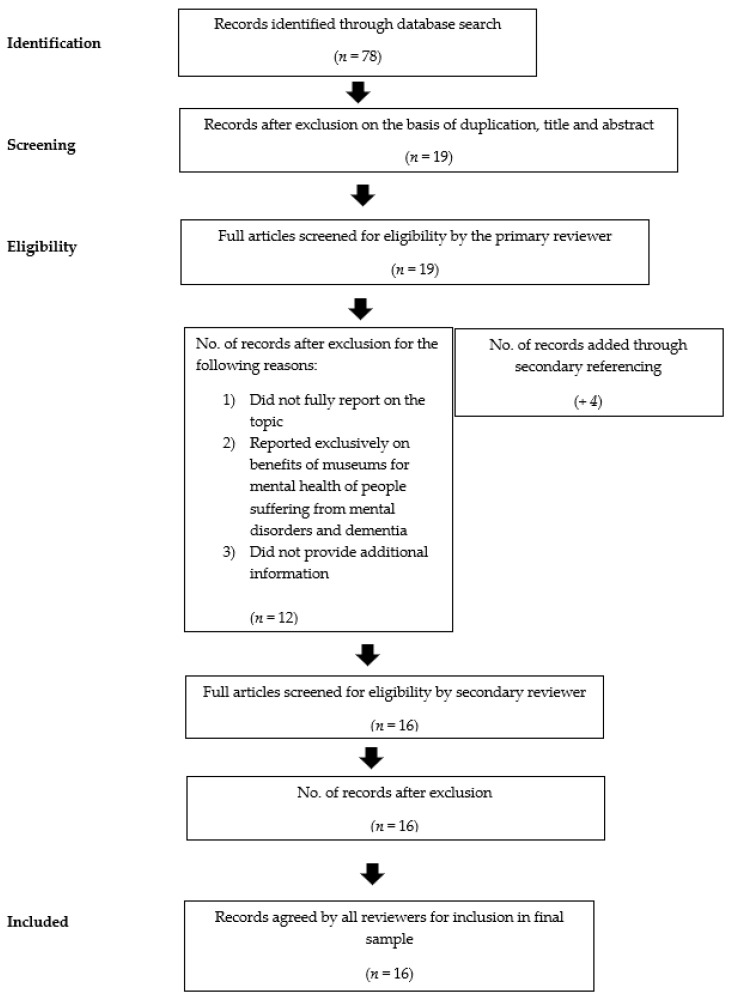
The PRISMA flow.

The PRISMA study selection process is illustrated in Figure 1.

After removing 43 duplicate references, the remaining 36 records were screened against the literature selection criteria. In the first stage of screening, papers were excluded based on their title and abstract if they did not clearly report on psychological wellbeing, eudemonia, wellbeing, mental health, or restorative experiences in museums. Papers were excluded based on title and abstract if they were entirely unrelated to wellbeing, not reporting on the topic of wellbeing in regard to museums, and if the review of the article was already included in the analysis. 

Following this, full texts were obtained for the remaining 19 records. At this point of analysis, we included records if they fully reported on the topic of interest, went beyond the benefits of museums for the mental health of marginalized populations, and provided additional information. Several records touched upon the topic of mental health in museums with an exclusive focus on people with mental health problems and dementia. Although we fully acknowledge the benefits that museum visitations may have, that topic has been covered by more research compared to the benefits that museum visitation may have for the psychological wellbeing of the general public, and was reviewed by Chatterjee and Noble in 2013. Thus, we aimed to review articles that deal to a larger extent with the psychological benefits of museum visits for the general public of visitors. However, we did include two records that focused on adult mental health service users and hospital patients because they explicitly studied psychological wellbeing and this greatly enriched our understanding of the psychological benefits of museum visitation. After applying these eligibility criteria and adding 4 records through secondary referencing, our final sample consisted of 16 records.

## 4. Results

The systematic review was carried out to synthesize the literature on psychological wellbeing in museums. We aimed to answer whether the museums could increase the psychological wellbeing of their visitors and how that might be achieved and measured. The Table 1 shows a list of records that were screened as being eligible to be included in the analysis. In the sections after the table, we combined the studies thematically to explore what is known about psychological wellbeing in museums: (1) Museum as a Restorative Environment, (2) Participation and Wellbeing, (3) Happier Sustainable Society, (4) Measuring Psychological Wellbeing, and (5) Examples of Psychological Wellbeing in Museums. 

### 4.1. Museum as a Restorative Environment

The idea that museums may have wellbeing benefits for their visitors appeared sometime in 1993, with the theory of restorative environment being applied to museums [14,20]. It was suggested that visitors do not come to museums solely for educational purposes and that museums may have a calming, restorative effect that helps visitors recover their cognitive and emotional effectiveness. That assumption is based on the Attention Restoration Theory, according to which, prolonged mental effort leads to directed attention fatigue (DAF). For example, work on a project, enjoyable or not, leads to DAF, which can have detrimental effects on the quality of life and wellbeing (distractibility, impatience, irritability, and impaired capacity to reason and plan). Although sleep may help, it is not sufficient, and it is necessary to rest directed attention when awake. This can be achieved in a restorative environment that allows for the presence of a wakeful but restful state. Kaplan, Bardwell and Slakter [14,20] empirically confirmed that museums could be a restorative environment, as they have the necessary properties that characterize such an environment. They conducted focus groups and a survey, but they did not measure DAF before and after the visitation, so the results, although highly suggestive and useful, do not offer definite conclusions. However, the researchers discovered that museums have the following restorative properties: (1) being away, (2) extent, (3) fascination, (4) compatibility. 

Being away entails an environment that is different to the usual, to allow for one to be away from his/her everyday concerns, which means that the environment must allow for one to spend some time in it. The third factor, and the most important factor of the restorative environment, fascination, involves the qualities of an environment that the visitor finds inherently interesting and that allows for one to use attention that does not require mental effort; hence, this allows for the visitor’s directed attention to rest. The fourth factor, compatibility, entails that the environment needs to support the visitor’s purpose in the museum, and not distract his/her attention [20]. Accordingly, “…the more the environment possesses these properties and the more strongly they are represented, the more restorative the environment is” [20]. Moreover, environments that have an aesthetic component tend to allow for a deeper restorative experience because they stimulate reflection, which works as an internal housekeeping that enables one to function with less of a need for directed attention in the future. Therefore, to obtain the psychological benefits of the restorative environment, the museums should promote reflection [14].

Chryslee [21] delved deeper into these properties in the museum and how they may be used to change people’s lives. In her article, she reviews studies that cover specific environmental cues and allow for the design of guidelines to create a restorative environment in the museums. However, the “being away” component is missing from her review because she assumed that museums inherently pose this factor. However, in the digital era, when museums can be visited virtually from the comfort of one’s home, being away is a component that needs to be explored. According to the author, to encourage visitor’s movement and curiosity (extent), museum designers should avoid using visual obstacles, large exhibitions, and guided paths, as they restrict visitor’s movement, and should use signage that is related to the exhibit [21]. Additionally, exploration may be encouraged using the following guidelines:

- Function of the object is determined by its location;
- Traffic in museums moves in clockwise direction;
- Similar objects have decreasing attraction for visitors;
- Objects on the left side of the room are looked at less;
- Exit attracts visitors away from nearby objects and signals the end of exploration and curiosity.

To help visitors feel comfortable and less intimidated, graphics that explain an object should answer the question: “What is this exhibit about”? Therefore, coherency can be achieved by encouraging observation through instructions, such as “look for…” and “notice…” [21]. 

Fascination can be achieved when visitors become cognitively involved with the exhibit and objects, which increases their viewing time. Viewing time can further be increased through colour, space, arrangement, and sound. Chryslee [21] reports the following findings, which are useful for museum experience designs:

- Colour brightness and saturation may be used to increase pleasure;
- Temperature should be set between 16 and 21 degrees Celsius;
- Increased intensity of light is pleasant, but glare or discontinuities in lighting are unpleasant;
- Sound should be simple and not variable;
- The more objects the museum has, the less time visitors will spend looking at each object;
- Label information should be provided;
- To increase the viewing time, similar objects should be added.

Compatibility, as the third restorative factor, entails feeling comfortable in the environment and content comprehension. Comfort can be achieved by following the same guidelines as for encouraging exploration, while content comprehension may be challenging, due to visitors often reporting not understanding the information in the museums. This is dependent on the visitors’ previous knowledge, which is difficult to for the museum designers to change. Chryslee [21] states the following useful design tools:

- The presence or absence of colour, distortion, style, and exaggerated scale signal the status of the object;
- Space should be organized in terms of boundaries, rhythms, tempos, and relationships among objects and between objects and people to communicate meaning;
- Object handling should be allowed, as well as enthusiastic expressions and energetic movement to stimulate participation.

Therefore, Chryslee [21] suggests that these environmental cues can be used to ensure the presence of restorative factors in the museums, which can then be used to create museums that change people’s lives. Furthermore, Packer and Bond [15] confirmed that museums have a restorative effect. Their research, which was conducted at four different museum-type environments (museum, gallery, aquarium, and botanic garden) using questionnaires, showed that gaining knowledge and reflecting were the most satisfying experiences in the museums and galleries, which added to the self-reported restorativness of the museum. The effect was stronger for frequent visitors than for first-time or occasional visitors. For infrequent visitors to experience the restorativness of the museum, museum experience designers should allocate more attention to providing a comfortable experience by controlling the temperature, lighting, noise and wayfinding, and providing rest areas. Furthermore, to enhance the restorative benefits, the visitors should be encouraged to reflect on their experience [15].

A newer study on restorativness of museums by Aeschbach et al. [32] investigated the effect of the visit’s duration on the museum’s restorativness. However, it should be noted that they did not directly measure museum restorativness, but rather the perceived restorativness of the environment. Their experimental between-subjects study found that participants who perceived their visit as being too long rated the resorativenss of the museum as lower compared to participants who perceived the length of their visit as either too short or ideal. Therefore, the subjective feeling of time duration (which may be affected by cognitive effort, attentional engagement, emotional state, intrinsic motivation, or lack of fascination) influenced the restorative benefits of the museum visit. Furthermore, participants who perceived their visit as being too long tended to be less art-experienced participants so it could be that they engaged in more cognitive effort, which affected their subjective visit duration. Furthermore, a short museum visit may serve as a micro-break, and museum designers are encouraged to create break areas (e.g., cafes, lounges) in the museum to increase the restorative benefits of the museum visit [32].

### 4.2. Participation and Wellbeing

Binne [22] observed the perception and experience of art within the museum through self-reported data using State Trait Anxiety Inventory and semi-structured interviews. She confirmed that even a simple viewing of art in the museums decreases anxiety and has a positive impact on wellbeing (the effect is stronger for frequent visitors) [22], but it was not possible to determine from the results whether the effect was achieved due to museum environment, the viewing of the artwork, or a combination of both. Although museums and artworks can have a clear wellbeing effect on the visitors without their engagement, researchers who explore the psychological wellbeing in museums tend to emphasize participation.

According to Fenton [25], museums can impact visitors’ wellbeing through museum learning activities, which are participative and exploratory, and hence involve social interaction. Throughout his review paper, where he also provides opinion and experience from the practice, Fenton [25] argues that participatory museums enable learning, which fosters wellbeing. Museums do this by “…engaging people in collaboration, fostering shared and individual experiences and emotions...”, while collections “…can facilitate participants to make connections with people and places, fostering feelings of inclusion in wider society”, and the use of arts “…enables participants to engage in methods of self-expression, exploring ideas and emotions whilst placing experience in the heart of learning”, which helps participants to “feel a sense of achievement and pride” [25] (p. 8). Therefore, to empower visitors, museums should offer opportunities for participation and interaction with others. 

One of the ways in which museums can encourage participation is by enabling object handling. Object handling typically involves visitors being able to explore the objects by touching them. Chatterjee, Vreeland and Noble [18] conducted 32 sessions of museum object handling with hospital patients and, based on the interviews and questionnaires, concluded that sessions had a positive impact on patient wellbeing through raising aspirations and developing their self-confidence. Furthermore, they observed the different ways the museum objects may be handled: stroking and petting, cautious handling, pulling the object close to oneself, working the object (opening and closing), absent-minded touching while looking elsewhere, exploratory touch, rough handling, and playful. During the object-handling sessions, patients were also asked questions about the object, which stimulated some of the patients to talk about their lives, regrets, and illness (personal/reminiscence), while others were more prone to use the objects-handling session for learning and being imaginative (impersonal/educational). Although they did not empirically confirm this, researchers concluded that such object-handling sessions can be beneficial for the general public, as well. For example, they could be used for a work-related training to enhance the self-confidence of the participants [18]. Moreover, Vogelpoel, Lewis-Holmes, Thomson and Chatterjee [24], used a mixed-methods research (surveys, focus groups, interviews and reflexive feedback), which involved volunteers ranging from students on museum, heritage, history, and medicine programmes to those studying programmes entirely unrelated to the topic. Further, adults who expressed their interest in participating were also included in the study. Hence, their sample reflected the volunteer population that would typically be interested in volunteering options within museums. The researchers found that object-handling sessions can also have a positive impact on the psychological wellbeing of the volunteer facilitators because facilitation of object handling develops their communication skills, reflexivity, creative engagement and the use of interpersonal skills. Thus, placing visitors in an active facilitation role enables community engagement and empowers their wellbeing [24]. 

Another study on museum object handling was conducted by Thomson, Lockyer, Camic and Chatterjee [27] in the museum setting with older adults (65–94). They created 10-week museum programmes of creative and socially interactive sessions (2 h each) that combined curator talks, behind-the-scenes tours, object handling and discussion, and arts activities inspired by the exhibits. They measured the psychological wellbeing of the participants before and after involvement in the programme using the Museum Wellbeing Measure for Older Adults (MWM-OA) and confirmed that the psychological wellbeing of the older adults significantly improved through learning and the acquisition of new skills and an enhanced sense of community and belonging. This was thoroughly explained by the researchers when they stated:

*“…when individuals interact with museums and collections, it is the intrinsic physical and material properties of the objects they encounter that trigger memories, projections, sensory, emotional, and cognitive associations. Museum objects may function as symbols for aspects of people’s lives such as identity, relationships, nature, society, and religion; these symbolic and meaning-making properties could account for their therapeutic potential; and the physical, cognitive and emotional interactions elicited by these multisensory object engagements have been identified as the unique value that museums can bring to public health interventions.”*

[27] (p. 34)

### 4.3. Happier Sustainable Society

One possibility for museums to collaborate, value the environment, and be diverse while simultaneously increasing the psychological wellbeing of their visitors is by offering a combined programme of arts and nature. Thomson, Morse, Elsden and Chatterjee [28] conducted a study with a combined arts-and-nature-based intervention, comprising engagement with horticulture, artmaking and museum collections on adult mental health service users. To do so, the study used exploratory sequential mixed methods design which included two phases. In phase 1, researchers investigated the views of participants through semi-structured interviews and diaries, while in Phase 2 participants completed the UCL Museum Wellbeing Measure pre-post programme. It was confirmed that such an intervention increased a sense of community, decreased social isolation, and supported self-esteem, thus having a strong impact on the quality of life and psychological wellbeing of the mental health service users. Although the effects were not empirically tested on the general public of visitors, museums with outdoor spaces should recognize their potential to offer a combined arts and nature experience that inherently involves creativity and multisensory engagement and can have transformative consequences for all the visitors by creating a happier, sustainable society [28].

Moreover, museum managers often evaluate the success of the museum based on the number of visitors and other short-term targets, while ignoring the central outcome they aim to achieve, which is providing experiences that are enjoyable, educational, and transformative. Hence, Thompson and Aked [23], in their review paper, which followed The Happy Museum project, argue that museums have a significant role in creating happier and sustainable society, but their evaluative focus needs to shift from counting visitors to counting the meaningful, rewarding, and empowering outcomes. Therefore, museums should promote social interaction and connections to other cultures and times, encourage visitors to be psychologically present with their attention focused on the here-and-now by using multisensory exhibits (and immersion), and encourage volunteering that directly influences the wellbeing of the individual. They should also adopt a more collaborative and facilitative approach. Thus, “…by encouraging happiness and wellbeing museums play a part in helping people live a good life without costing the earth” [23] (p. 8), and they can be the “high wellbeing, low carbon” leader by: using Five Ways to Wellbeing (Connect, Be active, Take notice, Keep learning, and Give), collaborating with others, valuing the environment, evaluating their success based on how their work affects the visitors, embracing the innovation (e.g., using digital tools), enabling social interaction, and finding their specific niche. These principles, although highly valuable, lack empirical support in the Thompson and Aked [23] review. 

### 4.4. Measuring Psychological Wellbeing

After designing a museum experience to enhance the psychological wellbeing of the visitors, it is necessary to measure that effect to evaluate the success of the intervention. We separated the following record from the rest of the records in the analysis because this is the only record that explains the necessary tools for the measurement of psychological wellbeing in the museums, rather than the effect of museums on wellbeing. It is important to note that there are several more tools for measuring psychological wellbeing (e.g., Ryff Psychological Wellbeing Scale); however, our literature review only yielded this measure, so it is the only tool analysed here. 

After a series of focus groups, surveys, trials of prototypes, and consultations with 32 UK museums, Thomson and Chatterjee [26] developed the Museum Wellbeing Toolkit for the museums to evaluate their wellbeing impact on the visitors. It was designed to measure psychological and subjective wellbeing. This toolkit “…can be used once by an individual service-user after one activity, before and after an activity to compare the impact with baseline data, to chart the progress of an individual across a series of sessions, and for a comparison of average scores across different groups in the same museum or across different museums” [26] (p. 58). It must be noted that this toolkit was developed by consultating only UK museums; hence, its applicability to different cultural contexts should be validated.

**Figure 2:**
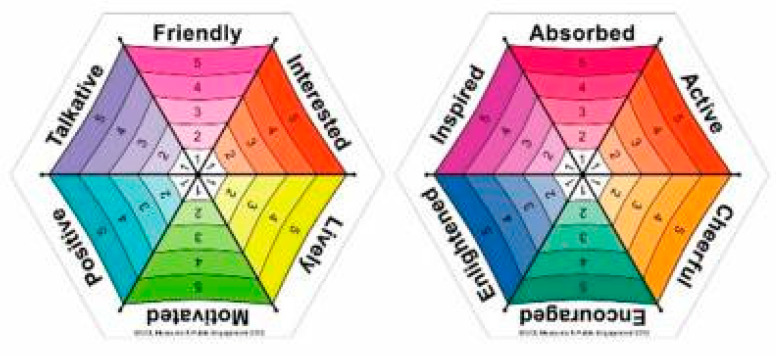

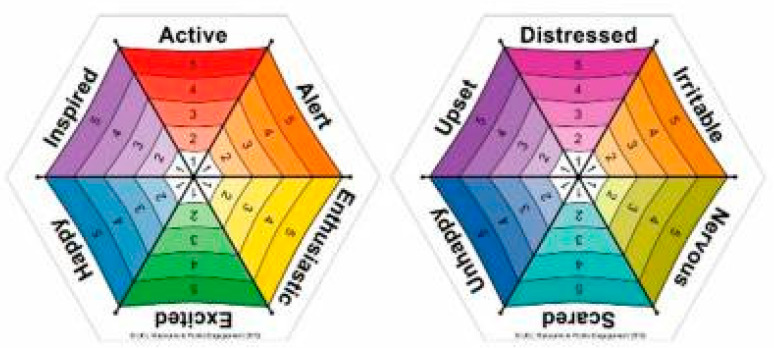
Museum Wellbeing Toolkit. (Thomson and Chatterjee, https://www.ucl.ac.uk/biosciences/culture-nature-health-research/ucl-creative-wellbeing-measures (accessed on 1 November 2022).

The toolkit contains three parts: Generic Wellbeing Questionnaire, Wellbeing Umbrellas, and Thoughts and comments section. The Generic Wellbeing Questionnaire can be filled out using a shorter version, which contains six statements, or using a longer version, which contains twelve statements. Following that, visitors use Wellbeing Umbrellas to determine their wellbeing, mood or emotions using a one-to-five rating scale. They do rate the extent of their wellbeing at that moment in time by circling the appropriate number. The four Wellbeing Umbrellas can be seen in Figure 2. The warm-colour umbrella represents the Generic Positive Wellbeing Umbrella, cool colours represent the Generic Negative Wellbeing Umbrella, rich colours represent a Positive Wellbeing Umbrella for Older Adults and fluorescent colours show a Positive Wellbeing Umbrella for Younger Adults [26]. 

### 4.5. Examples of Psychological Wellbeing in Museums

In this section, there are three examples of psychological wellbeing in museums that our search yielded and that may serve as an inspiration for museum experience designers. These three examples were thematically put together because they are an example of restorative, participative, and sustainable museums and wellbeing cultural policies in the UK, Australia, New Zealand, and Scotland. Specifically, the first two examples illustrate participation as one of the factors influencing psychological wellbeing in museums and the third example introduces an extremely important public policy, which may influence the governance and management of museums regarding wellbeing. 

#### 4.5.1. MindLab

Catalyst is a science discovery centre and independent museum, based in the UK, that explores the science and technology of the chemical industry and its impact on society. Before the COVID-19 lockdown, they partnered with mental health charity Mind Halton on the MindLab project, which was a science-inspired wellbeing project for local residents. They organized two programmes that lasted a few weeks. A typical session would start by naming the theme, such as “emotional pressures”. The counsellor would then lead a discussion using Cognitive Behavioural Techniques (CBT) and, after that, the same topic would be discussed from the science perspective. For example, the group would then engage in a workshop where they would explore the concept of pressure through chemical reactions. These programmes helped participants connect with others, reduced the feeling of social isolation, and increased their self-esteem [29]. Therefore, the MindLab project encourages museums to use CBT or other psychotherapeutic principles when designing experiences to enhance visitor’s psychological wellbeing by using museum objects as conversation starters or a means of exploring visitor’s psychological challenges through science and/or history.

#### 4.5.2. DIY Heritage Institutions: Australian Jazz Museum

The Australian Jazz Museum is a community based, do-it-yourself (DIY) museum focused on the preservation of jazz music and is almost exclusively founded and run by enthusiast volunteers. It is the so-called “third place” where people can gather and hang out. Cantillon and Baker [31] conducted interviews with the volunteers at the museum and discovered the museum has an abundance of wellbeing benefits for their volunteers, such as providing support networks, and raising the spirits of the participants, reducing stress, loneliness, and isolation. They stress the importance of a social network and collaboration in increasing the psychological wellbeing of the volunteers. Based on the example of the Australian Jazz Museum, museums should offer plenty of opportunities for community involvement, social interaction, and collaboration [31].

#### 4.5.3. Wellbeing Policy Framework: New Zealand and Scotland 

According to Lawler and Tissot [30], with the increasing competition for finances, museums need to prove their social role and community impact, so they have started to use “wellbeing” in public programme offerings and internal policymaking. Moreover, some nations, specifically New Zealand and Scotland, have begun to form wellbeing policy framework at the national level. For example, Jacinda Arden’s government delivered its first “wellbeing budget” in 2019, which impacted decision-making and stimulated museums to generate positive returns for society beyond economic growth. Similarly, the Scottish government placed the wellbeing in the core of National Performance Framework (NPF). Although the impact of such policies will take more time to develop compared to immediate indicators such as visitor numbers and sales revenues, these policies have propelled museums in New Zealand and Scotland to evaluate their success beyond the visit numbers and revenue, and emphasize the depth of learning and community engagement, shifting their focus to wellbeing economics [30].

## 5. Discussion

In order to explore psychological wellbeing in the museum context, we conducted a literature analysis using the PRISMA protocol to answer the following two research questions: (1) Can museums increase the visitor’s psychological wellbeing? (2) How can the museum experience be designed to enhance the psychological wellbeing of the visitors and how can that potentially be measured? Our review showed that museums can increase visitors’, but also museum experience facilitators’ (e.g., volunteers) psychological wellbeing. To achieve that effect, museum experiences should be designed to offer an attractive, comfortable (restorative), comprehensible, participative, innovative, and sustainable environment. Indeed, when policy frameworks that support wellbeing are present, the benefits for the visitors and other stakeholders are imminent because museums are encouraged to create experiences that have visitor’s wellbeing as a specific outcome. However, the impact of such policies will take more time to develop. Our review showed only one toolkit that specifically measures wellbeing in museums, called the Museum Well-being Toolkit.

### 5.1. Museum Design for Psychological Wellbeing

The results of the review are in line with theories from other academic fields, e.g., Packer and Bond’s [15] restorative effect of the museum is easily comparable with Pine and Gilmore’s [1] realms of experience. They propose four realms, according to the variety of consumer connection (absorption, immersion) and the form of the consumer’s participation (active or passive participation) in the experience. These are: esthetic, entertainment, education, and escape. While all four realms lead to an experience, not all of them offer the same intensity. Thus, an (a)esthetic experience, such as a visit to an art gallery, offers immersion but passive participation, since a visitor is mainly a passive observer. Watching a film is qualified as an entertaining experience, between absorption and passive participation. One absorbs the film’s narration while passively watching it. Educational experience, e.g., a culinary workshop, entails absorption and active experience since a participant absorbs the knowledge in an active way. Finally, an escapist experience can be considered the most intense type of experience, between immersion and active participation. Thus, acting in a theatre play entails identification with the character while actively performing [1]. Packer and Bond [15] confirmed that gaining knowledge and reflecting increase the restorativeness of the museum, which is in line with Pine and Gilmore’s educational experiences, absorption, and immersion. Pine and Gilmore suggest that a visit to an art gallery offers an aesthetic experience but is not on the highest intensity level in the experience realm. Along that line, the possibility of museums becoming restorative places may be increased by offering active participation, which would lead to escapist experiences. This may be a synonym for restorativeness and may eventually also lead to personal transformation.

The study also showed some concrete cues, which can be used in the experience design, which are also in line with Pine and Gilmore’s [1] experience design principles. Chatterjee, Vreeland and Noble [18] stress the direct relationship between object handling and psychological wellbeing. Further on, Chryslee [21] shows the importance of objects’ color, exaggerated scale, distortion and their handling in stimulating psychological wellbeing in museums. The importance of the sense of touch, as exhibited by Fenton [25], is seen in the advocacy for participation and interaction in the museums. In a similar way, Pine and Gilmore [1] underline the importance of engaging all five senses in order to achieve positive reactions by experience consumers. Senses are stimulated using cues such as colour, form and contrast; therefore, Chryslee [21], although mentioning only a few of them, is in line with Pine and Gilmore’s [1] engagement of the senses, as well as Thomson, Lockyer, Camic and Chatterjee’s [27] “multisensory object engagements” (p. 34), which show the value of museums for visitors’ wellbeing. Participation, as mentioned by Fenton [25], is also confirmed by Meijer-van Mensch and van Mensch [33], who opted for participative strategies in the museums. Participation through the engagement of senses and interpretation of the cues can elicit emotional reactions in visitors [11], which is important because positive emotions are one of the building blocks of psychological wellbeing and, when experienced, they stimulate participants to be more open in their interaction with others, which, in turn, enhances the sense of belonging (another building block of psychological wellbeing) [34]. 

### 5.2. Differences in Museum Visitors and Psychological Wellbeing

The fact that museum visits are transitory in nature poses a challenge for experience designers. Packer and Bond [15] claim that stronger restorative museum experiences take place for frequent visitors, which means that return visits should be encouraged. This has rationale in that repetition leads to experiences having memorability. Pine and Gilmore [1] also claim the greater memorability of experiences when applying their five experience design principles (theming, harmonizing impressions with positive cues, eliminating negative ones, supplying memorabilia, and engaging all the senses). This may lead to a greater understanding and meaning, eventually leading to restorativeness or transformation. If, however, museums focus on transitory visitors (e.g., tourists), it is difficult to encourage frequent repeated visits, but a possible recommendation could be repeating the main messages throughout the exhibition. For infrequent visitors, though, an engaging environment [15] may lead to enhanced restorative benefits.

Another challenge lies in approaching different visitors in terms of their previous experience with art. The findings of Aeschbach et al. [32] show that less art-experienced visitors usually perceive their visits as being too long, as a greater cognitive effort is required for them to draw any meaning. This can be compared with the results of Lindell and Mueller [35], who studied the psychological insights into art appreciation and concluded that art is valued as being more aesthetically pleasing in representational over abstract artworks; large and curved forms are valued over small and sharp ones; horizontal and vertical lines are valued over oblique ones. Along the same line, a moderate complexity of the artwork is preferred, since overly complex works may not reveal their meaning. As museum visitors are diverse, moderate levels of complexity and novelty would be optimal [35]. To engage the less art-experienced visitors, restorative effects could be enhanced by offering specific areas (e.g., cafes, lounges), as per Aeschbach et al. [32], but these should relate to the theme of the exhibition, as per Pine and Gilmore [1], to increase understanding and reveal meaning.

### 5.3. Measuring Museum Impact and Policy Framework

The meaning of the museum for the visitors and the community is specifically put forward by Thompson and Aked [23], who see it as being in a direct relationship with the individual. With that in mind, they advocate for an evaluation of the museum’s success based on the meaning and not the number of visitors. However, counting visitors and meaningful (transformative) visits are not mutually exclusive, but go hand in hand. Providing a meaning that leads to a quality transformative experience is important on a personal level (psychological wellbeing of an individual), but the better the experience, the more visitors museum attracts, which not only provides economic opportunities and financial benefits for the museum, but, at the same time, offers the opportunity to impact more people in terms of their own psychological wellbeing.

Apart from the specific design cues found in academic papers in this review study, the analyzed examples (MindLab, DIY heritage museums, wellbeing policies) put forward further techniques, which may be used. The first one concerns the use of psychotherapeutic principles in the museum experience design, as objects may serve as conversation starters. The second one reveals the importance of a social network and collaboration for museum stakeholders, from visitors to volunteers, which points to the need for a continuous development of the audience and to maintain a relationship with them. Finally, the public policy framework may be extremely stimulating for museums to redefine their role within the society and foster psychological, but, eventually, also collective wellbeing.

The implications of our findings are wide-ranging. Principles for the design of museum experiences that may stimulate the psychological wellbeing of visitors may be further applied to the design of exhibitions and galleries. Further, art activities in the museums, with the goal of education, may make use of the principles to create engaging and participative experiences that spark interest and personal growth. Such activities may be designed for school children. Moreover, art therapy can take place in the museums to empower people suffering from mental or physical issues and/or the general public dealing with traumatic experiences (e.g., COVID-19 or war). Museums can also be used for team-building activities for companies with object-handling sessions to elicit creativity and increase self-esteem. It is necessary to note that a negative implication of our findings may be seen in the instrumentalization of museums for the sake of health and wellbeing, which deprives museums of their inherent value. However, their important role in society should not be neglected. 

### 5.4. Limitations and Future Directions

Although (psychological) wellbeing is a hot topic across domains, a synthesized and holistic overview of what is known about psychological wellbeing regarding museums is lacking. Our review aimed to answer whether museums have the potential to enhance the psychological wellbeing and how that may be achieved through the experience’s design. We tried to offer a representative overview by using an extensive search of libraries worldwide, which included all-encompassing databases such as Scopus and Web of Science. However, we recognize that our search could be limited because we know of several papers that cover psychological wellbeing in cultural tourism (and some in museums), but the paper’s keywords do not include the paired words “psychological wellbeing (or eudemonia, or mental health, or wellbeing, or restorative)” and “museum” (for example, Jelinčić and Matečić [17]). Hence, future studies could explore psychological wellbeing in other cultural sectors and determine whether the knowledge and tools from those sectors are transferable to museums and vice versa. 

Apart from exploring the transferability of knowledge and tools to stimulate psychological wellbeing between the cultural sectors, future studies should make use of the physiological measures (e.g., electro-dermal analysis, heart rate, facial muscle activity, eye movement, and vascular activity) to directly measure the restorativeness of the museum, instead of relying solely on self-reported measures.

Furthermore, future studies could explore the differences in psychological wellbeing between different groups of visitors (e.g., age, gender), regardless of their experience with art, which was explored in studies mentioned in this review. For example, one museum might stimulate the psychological wellbeing of children, while another might stimulate the psychological wellbeing of adults. As wellbeing definitions differ between the cultures, it would be useful to explore the conceptual differences between interdependent and independent countries (east vs. west) in terms of how they study and understand psychological wellbeing in museums, and whether visitors from those countries expect psychological wellbeing as a museum visit outcome. For example, to use the basic east–west dichotomy, future studies could explore whether tourists from the east appreciate the psychological wellbeing benefits of the museums in the same way as tourists from the west. Such studies would help to enrich the multidisciplinary field of museums and wellbeing and offer opportunities for the creation of guidelines for designing museum experiences that cater to a broad range of visitors.

This review covered multiple museum design principles that may be used to stimulate visitor’s psychological wellbeing, but none of the studies that we found explored the competencies of the museum workers that are necessary to support psychological wellbeing in museums. It is evident that museum workers should be trained in experience design and have basic knowledge of the psychological principles of eliciting emotion and social inclusion. This, however, may be an avenue for future studies.

Despite our research showing that museums play a central role in enhancing the psychological wellbeing of their visitors and other stakeholders, there is a substantial lack of studies that explicitly study the effect of museums on the psychological wellbeing of the general public (e.g., children, young adults, adults, older people, tourists). Most of the studies that our search uncovered focused on the marginalized population (e.g., hospital patients, mental health service users). However, as the remaining publications that were selected as being eligible for this research showed the clear psychological benefits that museum visitation can have on the general public, it is necessary to explore this field in more depth to create museum activities and design a museum environment that empowers visitors. Our research showed that this can be achieved by creating a restorative, comfortable environment that encourages participation via multisensory engagement, object handling, visitor interaction and volunteering, whilst preserving the environment. Therefore, museums, especially when encouraged by the policy framework, have the potential to help people and society to thrive. As wellbeing public policies have been rather rare to date, future research may explore the effects of the existing ones to provide recommendations for new developments on this topic.

## 6. Conclusions

**Figure 3:**
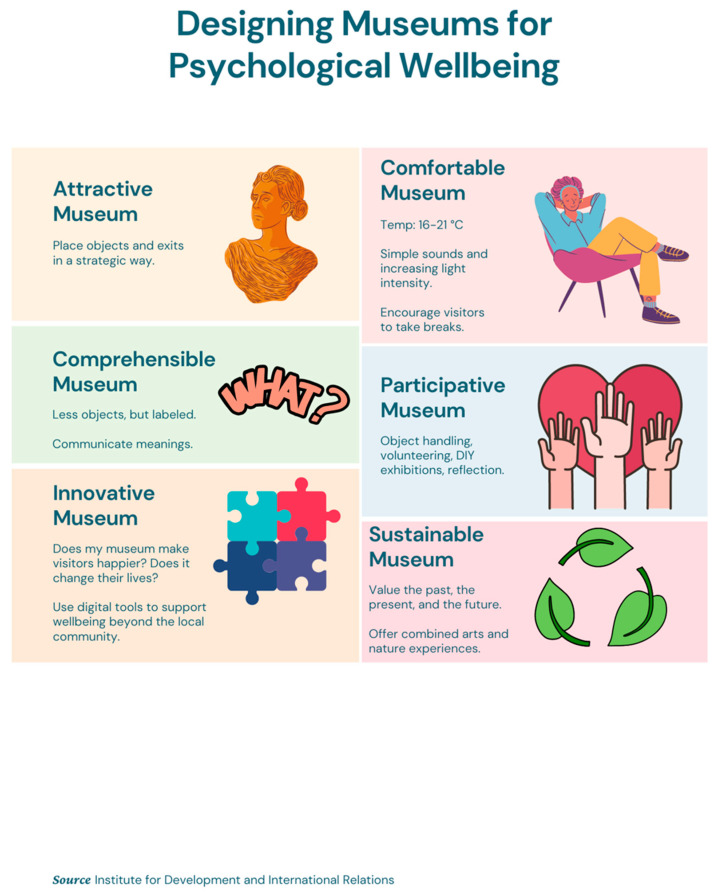
Designing Museums for Psychological Wellbeing.

Finally, to synthesize the findings and offer an easy-to-read toolkit for museum experience designers who want to create experiences that can enhance psychological wellbeing in museums, we provide the following list of guidelines, separated into six overarching themes that museums should follow. Additionally, we provide Figure 3, which may be printed out or used as a reminder of the guidelines.

1. 1. Attractive Museums
  - Place objects in a strategic way (e.g., similar objects should be placed away from each other, objects should be placed on the right side of the room because movement occurs in a clockwise direction across the room).
  - Be aware that exits attract visitors away from nearby objects and signal the end of exploration and curiosity.
2. Comfortable Museum
  - Use colour and brightness to affect pleasure.
  - Set museum temperature between 16 and 21 degrees Celsius.
  - Use increasing light intensity without discontinuities in lighting.
  - Use the simple sound of decreased loudness with less variability.
  - Encourage visitors to take regular breaks by providing, e.g., cafes, lounge areas.
3. Comprehensible Museum
  - Place fewer (but related) objects in the room to increase the viewing time.
  - Provide label information.
  - Use colour, distortion, style, and exaggerated scale to communicate the status of the object.
  - Organize space in terms of boundaries, rhythms, tempos, and relationships among objects and between objects and people to communicate meanings and purposes.
4. Participative Museum
  - Allow for object handling.
  - Allow for enthusiastic expressions and energetic movement to stimulate participation.
  - Encourage visitors to reflect on their experience.
  - Offer opportunities for collaboration and interaction between the visitors.
  - Offer volunteering opportunities.
  - Enable the creation of DIY exhibitions to engage the community.
5. Innovative Museum
  - Evaluate your success based on how your work affects your visitors (Does it make them happier? Does it change their lives?).
  - Use digital tools to support well-being beyond the local community.
  - Find your niche.
  - Connect the psychotherapeutic principle and objects to start conversations about psychological challenges.
6. Sustainable Museum
  - Value the environment, the past, the present and the future.
  - Offer a combined arts and nature experience by utilizing the museum’s outdoor space or nearby parks.

## Figures and Tables

**Table 1:** Records included in the analysis.

Authors	Title	Year
Kaplan, Bardwell and Slakter	The Restorative Experience as a Museum Benefit	1993 [14]
Kaplan, Bardwell and Slakter	The Museum as a Restorative Environment	1993 [20]
Chryslee	Creating Museums That Change People’s Lives	1995 [21]
Chatterjee, Vreeland and Noble	Museopathy: Exploring the Healing Potential of Handling Museum Objects	2009 [18]
Binne	Does Viewing Art in the Museum Reduce Anxiety and Improve Wellbeing?	2010 [22]
Packer and Bond	Museums as Restorative Environments	2010 [15]
Thompson and Aked	The Happy Museum: A tale of how it could turn out all right	2011 [23]
Vogelpoel, Lewis-Holmes, Thomson and Chatterjee	Touching Heritage: Community Health and Wellbeing Promotion through Sustainable and Inclusive Volunteer Programming in the Museums Sector	2013 [24]
Fenton	Museums, participatory arts activities and wellbeing	2013 [25]
Thomson and Chatterjee	Measuring the impact of museum activities on well-being: developing the Museum Well-being Measures Toolkit	2015 [26]
Thomson, Lockyer, Camic and Chatterjee	Effects of a museum-based social prescription intervention on quantitative measures of psychological wellbeing in older adults	2017 [27]
Thomson, Morse, Elsden and Chatterjee	Art, nature and mental health: assessing the biopsychosocial effects of a ‘creative green prescription’ museum programme involving horticulture, artmaking, and collections	2020 [28]
French, Lunt and Pearson	The MindLab Project. Local Museums Supporting Community Wellbeing Before and After UK Lockdown	2020 [29]
Lawler and Tissot	Preserving the intangible and immeasurable: exploring wellbeing frameworks in the museum context	2021 [30]
Cantillon and Baker	DIY Heritage Institutions as Third Places: Caring, Community and Wellbeing Among Volunteers at the Australian Jazz Museum	2022 [31]
Aeschbach et al.	Less is more: The Effect of Visiting Duration on the Perceived Restoratives of Museums	2022 [32]

## Data Availability

No new data were created or analyzed in this study. Data sharing is not applicable to this article.

## References

[1] Pine B.J., Gilmore J.H. (1998). Welcome to the experience economy. Harv. Bus. Rev..

[2] McKercher B., Wong D.Y.Y. (2004). Understanding tourism behavior: Examining the combined effects of prior visitation history and destination status. J. Travel Res..

[3] Kotler N., Kotler P. (2000). Can Museums be All Things to All People?: Missions, Goals, and Marketing’s Role. Mus. Manag. Curatorship.

[4] Packer J. (2008). Beyond learning: Exploring visitors’ perceptions of the value and benefits of museum experiences. Curator Mus. J..

[5] Camic P.M., Chatterjee H.J. (2013). Museums and art galleries as partners for public health interventions. Perspect. Public Health.

[6] Diener E. (1984). Subjective well-being. Psychol. Bull..

[7] Ryff C.D. (1989). Happiness is everything, or is it? Explorations on the meaning of psychological well-being. J. Personal. Soc. Psychol..

[8] Ruggeri K., Garcia-Garzon E., Maguire A., Matz S., Huppert F.A. (2020). Well-being is more than happiness and life satisfaction: A multidimensional analysis of 21 countries. Health Qual. Life Outcomes.

[9] Smith M.K., Diekmann A. (2012). Tourism and wellbeing. Ann. Tour. Res..

[10] Diener E., Seligman M.E.P. (2004). Beyond Money: Toward an Economy of Well-Being. Psychol. Sci. Public Interest.

[11] Jelinčić D.A., Šveb M. (2021). Visual Stimuli Cues with Impact on Emotions in Cultural Tourism Experience Design. Acta Tur..

[12] Pine B.J., Gilmore J.H. (2013). The experience economy: Past, present and future. Handbook on the Experience Economy.

[13] European Travel Commission (Annual Report 2016). https://etc-corporate.org/uploads/Annual-Report-2016_final.pdf.

[14] Kaplan S., Bardwell L.V., Slakter D.B. (1993). The Restorative Experience as a Museum Benefit. J. Mus. Educ..

[15] Packer J., Bond N. (2010). Museums as restorative environments. Curator Mus. J..

[16] Mastandrea S., Fagioli S., Biasi V. (2019). Art and psychological well-being: Linking the brain to the aesthetic emotion. Front. Psychol..

[17] Jelinčić D.A., Matečić I. (2021). Broken but Well: Healing Dimensions of Cultural Tourism Experiences. Sustainability.

[18] Chatterjee H., Vreeland S., Noble G. (2009). Museopathy: Exploring the healing potential of handling museum objects. Mus. Soc..

[19] Chatterjee H., Noble G. (2016). Museums, Health and Well-Being.

[20] Kaplan S., Bardwell L.V., Slakter D.B. (1993). The museum as a restorative environment. Environ. Behav..

[21] Chryslee G.J. (1995). Creating museums that change people’s lives: Operationalizing the notion of restorative environments. J. Mus. Educ..

[22] Binnie J. (2010). Does viewing art in the museum reduce anxiety and improve wellbeing?. Mus. Soc. Issues.

[23] Thompson S., Aked J. (2011). The Happy Museum: A Tale How It Could All Turn All Right.

[24] Vogelpoel N., Lewis-Holmes B., Thomson L., Chatterjee H. (2013). Touching heritage: Community health and wellbeing promotion through sustainable and inclusive volunteer programming in the museums sector. Int. J. Incl. Mus..

[25] Fenton H. (2013). Museums, participatory arts activities and wellbeing. Teach. Lifelong Learn..

[26] Thomson L.J., Chatterjee H.J. (2015). Measuring the impact of museum activities on well-being: Developing the Museum Well-being Measures Toolkit. Mus. Manag. Curatorship.

[27] Thomson L.J., Lockyer B., Camic P.M., Chatterjee H.J. (2018). Effects of a museum-based social prescription intervention on quantitative measures of psychological wellbeing in older adults. Perspect. Public Health.

[28] Thomson L.J., Morse N., Elsden E., Chatterjee H.J. (2020). Art, nature and mental health: Assessing the biopsychosocial effects of a ‘creative green prescription’ museum programme involving horticulture, artmaking and collections. Perspect. Public Health.

[29] French J., Lunt N., Pearson M. (2020). The MindLab project. local museums supporting community wellbeing before and after uk lockdown. Mus. Soc..

[30] Lawler N., Tissot A. (2021). Preserving the intangible and immeasurable: Exploring wellbeing frameworks in the museum context. J. Inst. Conserv..

[31] Cantillon Z., Baker S. (2022). DIY heritage institutions as third places: Caring, community and wellbeing among volunteers at the australian jazz museum. Leis. Sci..

[32] Aeschbach V.M., Schipperges H., Braun M.A., Ehret S., Ruess M., Sahintuerk Z., Thomaschke R. (2022). Less Is More: The Effect of Visiting Duration on the Perceived Restorativeness of Museums. https://psycnet.apa.org/record/2022-59600-001.

[33] Meijer-van Mensch L., van Mensch P. (2013). Introduction. Participative Strategies in Collecting the Present.

[34] Lambert L., Lomas T., van de Weijer M.P., Passmore H.A., Joshanloo M., Harter J., Ishikawa Y., Lai A., Kitagawa T., Chen D. (2020). Towards a greater global understanding of wellbeing: A proposal for a more inclusive measure. Int. J. Wellbeing.

[35] Lindell A.K., Mueller J. (2011). Can science account for taste? Psychological insights into art appreciation. J. Cogn. Psychol..

